# TMEM16A drives renal cyst growth by augmenting Ca^2+^ signaling in M1 cells

**DOI:** 10.1007/s00109-020-01894-y

**Published:** 2020-03-18

**Authors:** Ines Cabrita, Björn Buchholz, Rainer Schreiber, Karl Kunzelmann

**Affiliations:** 1 Institut für Physiologie, Universität Regensburg, Universitätsstraße 31, 93053 Regensburg, Germany; 2 Department of Nephrology and Hypertension, University of Erlangen-Nuremberg, Erlangen, Germany

**Keywords:** ADPKD, Renal cysts, TMEM16A, Anoctamin 1, Ca^2+^-activated Cl^−^ channel

## Abstract

**Abstract:**

Polycystic kidney disease (PKD) leads to continuous decline of renal function by growth of renal cysts. Enhanced proliferation and transepithelial chloride secretion through cystic fibrosis transmembrane conductance regulator (CFTR) and Ca^2+^-activated TMEM16A Cl^−^ channels is thought to cause an increase in cyst volume. Recent work shows the pro-proliferative role of the Ca^2+^ activated Cl^−^ channel TMEM16A (anoctamin 1), and demonstrates the essential contribution of TMEM16A to CFTR-dependent Cl^−^ secretion. The present data demonstrate an increase in intracellular Ca^2+^ ([Ca^2+^]i) signals and Cl^−^ secretion by TMEM16A, in renal collecting duct principle cells from dog (MDCK) and mouse (M1) as well as primary tubular epithelial cells from PKD1−/− knockout mice. M1 organoids proliferated, increased expression of TMEM16A, and secreted Cl^−^ upon knockdown of endogenous polycystin 1 or 2 (PKD1,2), by retroviral transfection with shPKD1 and shPKD2, respectively. Knockdown of PKD1 or PKD2 increased basal intracellular Ca^2+^ levels and enhanced purinergic Ca^2+^ release from endoplasmic reticulum. In contrast, ryanodine receptors were found not to be expressed in mouse renal epithelial cells and caffeine had no effects on [Ca^2+^]i. Ca^2+^ signals, proliferation, and Cl^−^ secretion were largely reduced by knockdown or blockade of TMEM16A. TMEM16A may be therefore important for enhanced Ca^2+^ release from IP_3_-sensitive Ca^2+^ stores in polycystic kidney disease.

**Key messages:**

• ADPKD leads to continuous decline of renal function by growth of renal cysts.

• Knockdown of PKD1 or PKD2 increases TMEM16A expression.

• TMEM16A enhanced intracellular Ca^2+^ signals, Cl^−^ secretion, and proliferation.

• TMEM16A contributes to cyst growth in ADPKD.

**Electronic supplementary material:**

The online version of this article (10.1007/s00109-020-01894-y) contains supplementary material, which is available to authorized users.

## Introduction

Frequent autosomal dominant polycystic kidney disease (ADPKD) accounts for 5–10% of end-stage renal disease [1]. ADPKD is characterized by continuous cyst enlargement over time, leading to compression of adjacent healthy parenchyma [2]. ADPKD is caused by mutations in PKD1 (polycystin 1) or PKD2 (polycystin 2), but the underlying complex molecular events leading to continuous cyst growth are still poorly understood [3]. In normal renal epithelial cells, PKD1 and PKD2 appear to be located in the primary cilium, a single antenna-like protrusion of the plasma membrane, where they form a complex of receptor and Ca^2+^ influx channel [4]. Ca^2+^ ions are more concentrated within the primary cilium compared to the cytoplasm; however, Ca^2+^ signals generated within the cilium may occur independent of cytoplasmic Ca^2+^ signaling [5]. Loss of the primary cilium or loss of PKD1/PKD2 function leads to relocation of the polycystins to plasma membrane and endoplasmic reticulum, with the consequence of disturbed intracellular Ca^2+^ signaling [6].

We reported earlier an upregulation of the Ca^2+^ activated chloride channel TMEM16A (anoctamin 1) in polycystic kidney disease. TMEM16A enables calcium-activated chloride secretion that supports expansion of renal cysts and probably proliferation of the cyst-forming epithelium [7]. Remarkably, primary cilia present in terminally differentiated naïve cells or in non-proliferating cells in culture, contain TMEM16A as well as the paralogous proteins TMEM16F and TMEM16K [8–10]. Loss of expression of TMEM16A was shown to compromise ciliary genesis and decreased length of the primary cilium and of motile cilia [8, 11, 12].

In the presence of TMEM16A, basal and agonist-induced Ca^2+^ levels are increased [13, 14]. TMEM16A was shown to couple to inositol 1,4,5-trisphosphate (IP_3_) receptors [13, 14] and different TRP Ca^2+^ influx channels [15]. TMEM16A enhances ER Ca^2+^ store release by tethering the ER to the membrane localized receptor signaling complex. As a result, transmembrane signaling, fluid secretion, or general cellular properties like proliferation, migration, or volume regulation are affected. We examined in the present study whether TMEM16A contributes to disturbed Ca^2+^ signaling observed in ADPKD. We further asked whether TMEM16A-related changes in Ca^2+^ signaling affect proliferation and fluid secretion. We found upregulation of TMEM16A with the loss of PKD1 or PKD2 expression in M1 collecting duct cells. In M1 cells, TMEM16A supported Ca^2+^ store release, cell proliferation, and fluid secretion and thereby contributed to cyst growth.

## Results

### TMEM16A augments fluid secretion by increase in intracellular Ca^2+^

**Fig. 1:**
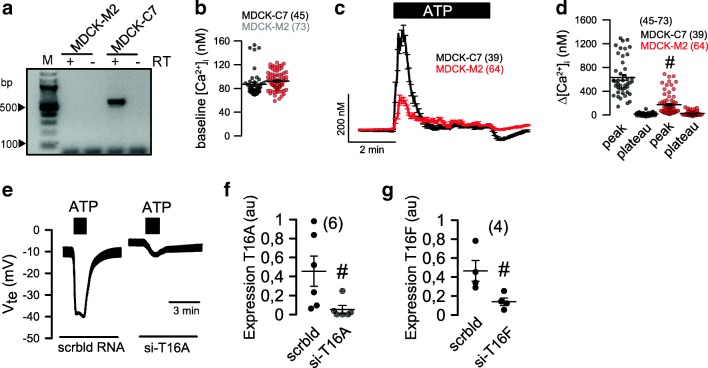
TMEM16A augments Ca^2+^ signaling and ion transport in MDCK cells. **a** RT-PCR indicating expression of TMEM16A in MDCK-C7 cells but not in MDCK-M2 cells. **b** Summary of basal Ca^2+^ levels in MDCK-C7 and MDCK-M2 cells. **c**, **d** Assessment of intracellular Ca^2+^ using the Ca^2+^ sensor Fura2. ATP or UTP (both 100 μM) increased intracellular peak and plateau Ca^2+^ in MDCK-C7 and MDCK-M2 cells. **e** Original recordings and summary of ATP or UTP induced transepithelial voltages in MDCK-C7 cells and effect of TMEM16A-knockout. **f**, **g** Effect of siRNA on expression of TMEM16A and TMEM16F, respectively, as assessed by quantitative RT-PCR. Mean ± SEM (number of cells measured). #Significant difference when compared to MDCK-C7 and scrambled, respectively

**Fig. 2:**
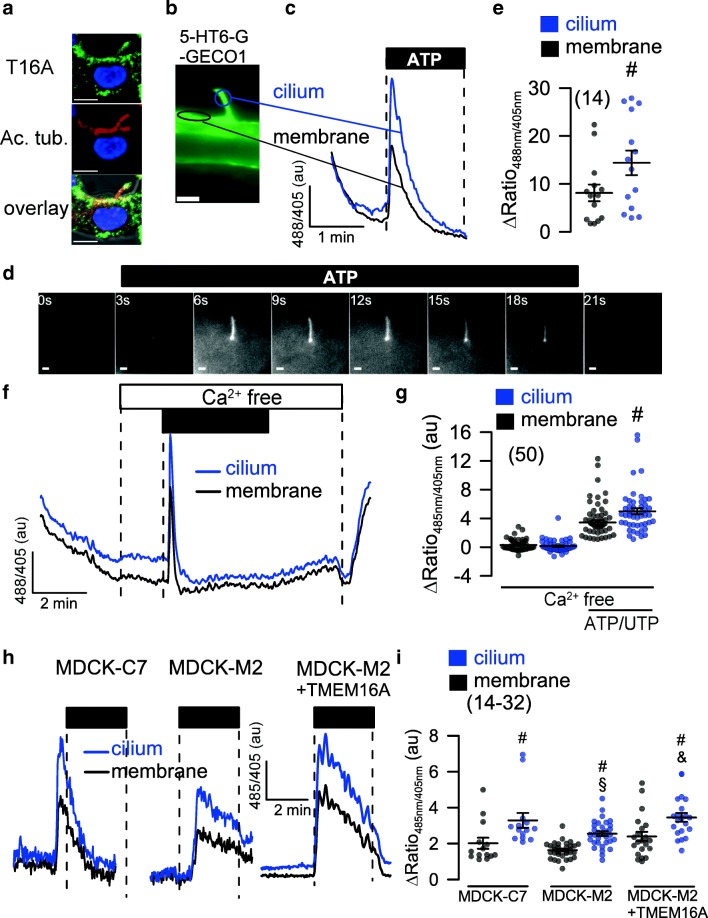
Role of TMEM16A in plasma membrane and primary cilium of MDCK cells. **a** TMEM16A (green), acetylated tubulin (red), and overlay showing expression of TMEM16A in primary cilium and plasma membrane of a naïve renal epithelial cell. **b** Ca^2+^ sensor 5-HT6-G-GECO1 expressed in the primary cilium and near plasma membrane allowing measurement of Ca^2+^ in both compartments. **c**–**e** Original recordings and summary of Ca^2+^ signals elicited by stimulation with ATP or UTP (both 100 μM) in primary cilium and near plasma membrane. Bars = 1 μm. **f**, **g** Increase of intracellular Ca^2+^ in the absence of extracellular Ca^2+^. **h**, **i** Comparison of purinergic Ca^2+^ increase in MDCK-C7 (expressing TMEM16A) and MDCK-M2 (not expressing TMEM16A). Expression of TMEM16A in the M2 clone increased the ATP-induced Ca^2+^ signal in both cilium and plasma membrane. Bars = 2 μm. Mean ± SEM (number of cells measured). ^#^Significant difference when compared to membrane (*p* < 0.05; unpaired *t* test). ^§^Significant difference when compared to MDCK-C7 (*p* < 0.05; unpaired *t* test)

We demonstrated earlier the impact of TMEM16A on fluid secretion and cyst growth in a MDCK cyst model and in embryonic kidney cultures [7]. MDCK cells derived from dog principle cells exist as a MDCK-C7 clone expressing TMEM16A, and as a MDCK-M2 clone, lacking expression of TMEM16A (Fig. 1a). The Ca^2+^ sensor Fura2 showed a remarkable increase in intracellular Ca^2+^ when MDCK-C7 cells were stimulated with the purinergic agonists ATP or UTP (Fig. 1b–d). In contrast, MDCK-M2 cells lacking expression of TMEM16A showed a much reduced Ca^2+^ response upon purinergic stimulation. In TMEM16A-expressing MDCK-C7 cells, a pronounced Cl^−^ secretion was activated by ATP/UTP, which was potently inhibited by small interfering RNA (siRNA)-knockout of TMEM16A (Fig. 1e) [7]. In contrast, Cl^−^ secretion is largely reduced in MDCK-M2 cells, as reported earlier [9]. Moreover, because siRNA-knockdown of TMEM16F did not affect Ca^2+^ activated Cl^−^ currents, the data suggest that TMEM16A is the Ca^2+^-activated Cl^−^ channel in MDCK-C7 cells, similar to other tissues (Fig. 1f, g) [9]. TMEM16A was found to be expressed in plasma membrane and primary cilium of MDCK and naïve renal tubular epithelial cells [8, 10] (Fig. 2a). Ca^2+^ changes in primary cilium and near the plasma membrane were measured using 5-HT6-G-GECO1 (kindly provided by Prof. Takanari Inoue, Johns Hopkins University, Baltimore, USA; Fig. 2b). A Ca^2+^ rise in both cilium and near plasma membrane was detected upon purinergic stimulation with ATP or UTP (Fig. 2c–e). Importantly, peak increase in [Ca^2+^]_i_ (store release) was similar in plasma membrane and cilium also in the absence of extracellular Ca^2+^ (Fig. 2f, g). Purinergic Ca^2+^ rise was larger in the primary cilium than close to the plasma membrane, but otherwise qualitatively similar. It was attenuated in MDCK-M2 cells lacking expression of TMEM16A (Fig. 2h, i). Moreover, overexpression of TMEM16A in MDCK-M2 increased the ATP-induced Ca^2+^ signal in both cilium and plasma membrane. These results are in line with the previously reported role of TMEM16 proteins in Ca^2+^ signaling [14].

### Loss of PKD1 or PKD2 induces Cl^−^ secretion in M1 renal organoids

**Fig. 3:**
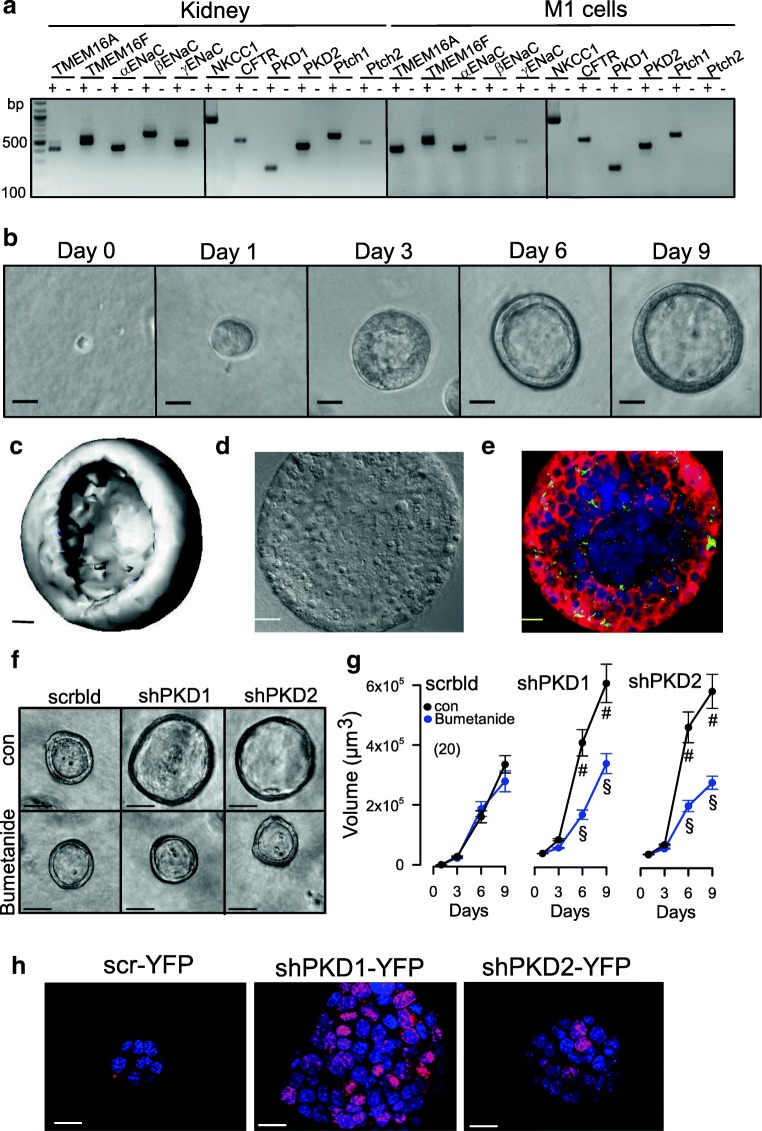
M1 renal organoid and cyst model. **a** RT-PCR analysis of mRNA expression of ion channels and receptors in mouse kidney and M1 collecting duct cells: TMEM16A, TMEM16F, αβγ-ENaC, NKCC1, PKD1, PKD2, and the receptors patched 1,2 (Ptch1,2). Similar expression patterns were found in mouse kidney and M1 cells. ± indicate presence/absence of reverse transcriptase. **b** Time-dependent development of renal organoids in Matrigel (*n* = 20). Bars = 20 μm. **c** Reconstructed 3D image from a renal M1 organoid with a view inside the organoid. Bar = 20 μm. **d**, **e** Differential interference contrast (DIC) image and immunocytochemistry of a cross-section of an organoid. Green, primary cilia; red, CFTR; blue, DAPI. Bars = 20 μm. **f**, **g** Increase of the volume of M1 organoids by shRNA-knockdown of PKD1 or PKD2. The presence of the NKCC1-inhibitor bumetanide (100 μM) did not change the size of control organoids (treated with scrambled RNA; scrbld), but inhibited further enlargement by shRNA-knockdown of PKD1 or PKD2, indicating fluid secretion upon knockdown of PKD1,2. Bars = 50 μm. **h** Increase of proliferative activity in shPKD1/shPKD2 organoids as indicated by Ki-67 staining. Bars = 20 μm. Mean ± SEM (number of organoids measured). ^#^Significant difference when compared with scrambled (*p* < 0.05; unpaired *t* test). ^§^Significant difference when compared to absence of bumetanide (*p* < 0.05; unpaired *t* test)

**Fig. 4:**
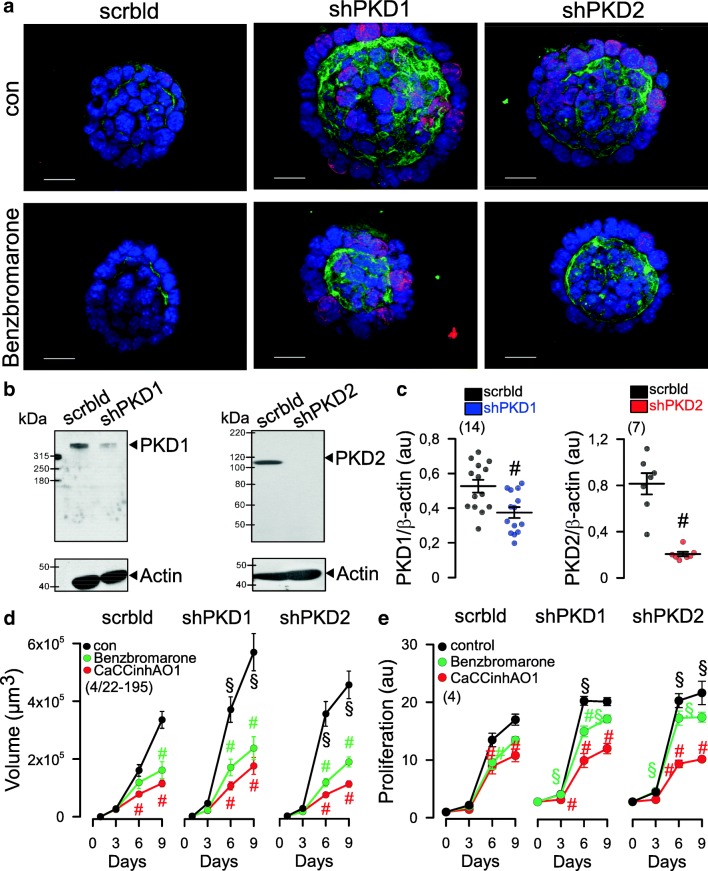
Increased expression of TMEM16A, proliferation and organoid growth by knockdown of PKD1 or PKD2. **a** Knockdown of both PKD1 or PKD2 induced cell proliferation (Ki-67 staining; red), enhanced expression of TMEM16A (green) and growth of M1 organoids. **b**, **c** Western blot and quantification of mRNA by semi-quantitative RT-PCR indicates significant knockdown of PKD1 and PKD2 by small hairpin (sh) RNA. Western blots were performed *n* = 14 and *n* = 7 times, respectively, and as indicated, and a knockdown by 61% and 92%, respectively, was achieved. Protein loading was not normalized among the lanes. **d**, **e** Increase in cyst volume and proliferation upon knockdown of PKD1/PKD2, and inhibition by 5 μM benzbromarone or CaCCinh172 [34]. Bars = 20 μm. Ki67 was quantified by measuring fluorescence intensity. Mean ± SEM (number of organoids measured). ^#^Significant difference when compared control (*p* < 0.05; ANOVA). ^§^Significant difference when compared to scrambled (*p* < 0.05; ANOVA)

We further examined the role of TMEM16A and other PKD-associated proteins for cyst growth, cell proliferation, and Ca^2+^ signaling using a 3D culture model. To that end, M1 mouse collecting duct cells were analyzed for expression of the relevant proteins polycystin (PKD1, PKD2), TMEM16A, TMEM16F, CFTR, NKCC1, and αβγ-ENaC (Fig. 3a). Cells were grown in a collagen/Matrigel matrix and readily formed spherical renal organoids (Fig. 3b, c). The cells appeared highly differentiated and produced primary cilia (Fig. 3d, e). Importantly, M1 renal organoids do not seem to secrete fluid, because the NKCC1 inhibitor bumetanide did not interfere with the formation and growth of the organoid (Fig. 3f, g). However, organoids expressed epithelial Na^+^ channels and increased their volume when grown in amiloride (not shown). Knockdown of PKD1 or PKD2 increased the organoid volume, and this increase in volume was inhibited by bumetanide, indicating activation of ion secretion upon knockdown of polycystins and induction of a cystic phenotype (Figs. 3f, g and 4c).

### Enhanced secretion and proliferation in PKD requires TMEM16A

**Fig. 5:**
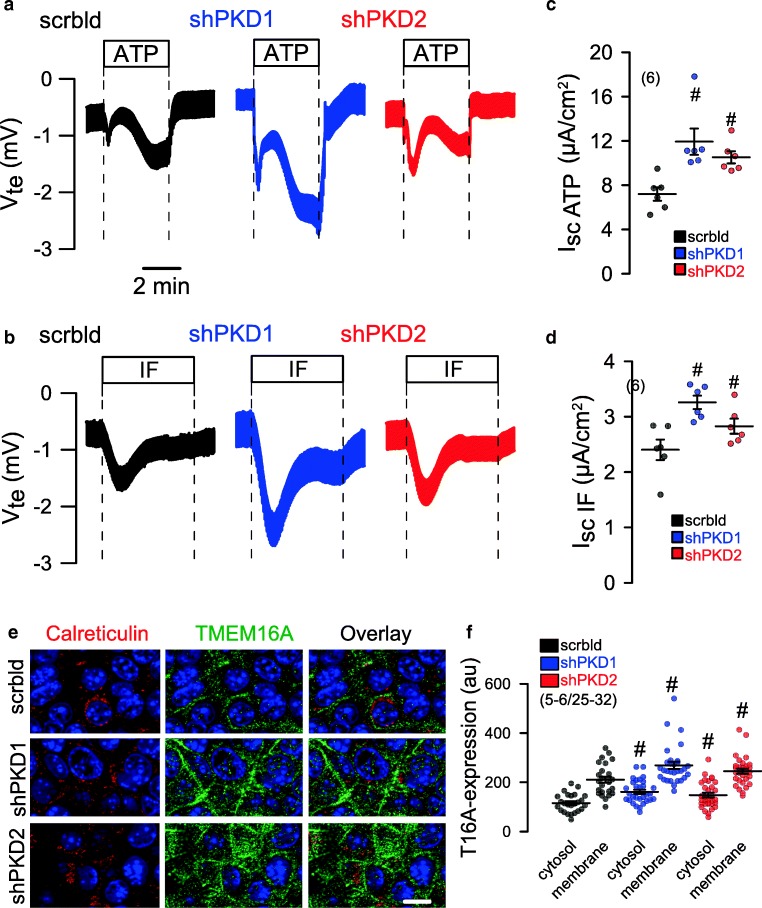
Induction of Cl^−^ secretion by knockdown of PKD1 or PKD2. **a**, **c** Original Ussing chamber recordings of polarized M1 cells grown permeable supports (2D culture). Enhanced Cl^−^ secretion by luminal stimulation with ATP (100 μM) or IF (100 μM IBMX and 2 μM forskolin) upon shRNA-knockdown of PKD1 or PKD2. **b**, **d** Summaries for calculated equivalent basal short-circuit currents (I_sc_) and I_sc_ activated by ATP and forskolin/IBMX, respectively. **e**, **f** Co-staining of TMEM16A (green) and calreticulin. Knockdown of PKD1 or PKD2 upregulated TMEM16A in plasma membrane and cytosol. No colocalization with calreticulin was observed. Bar = 20 μm. Mean ± SEM (number of organoids measured). ^#^Significant difference when compared scrambled (*p* < 0.05; ANOVA)

A hallmark of renal cysts is the upregulation of proliferation [16]. TMEM16A is well known to cause cell proliferation, Cl^−^ secretion and proliferation. The proliferation marker Ki-67 demonstrated a strong upregulation of proliferation of M1 renal organoids upon knockdown of PKD1 or PKD2 (Fig. 4a). The effect of short hairpin RNA (shRNA)-PKD1 and shRNA-PKD2 was analyzed by Western blotting/densitometry and indicated a knockdown by 61% and 92%, respectively. In addition, significant suppression of messenger RNA (mRNA) for PKD1 and PKD2 was demonstrated by semi-quantitative RT-PCR (Fig. 4b, c). Attenuation of expression of PKD1 or PKD2 and consecutive rise in cell proliferation was paralleled by a strong increase in TMEM16A expression (Fig. 4a, green staining). The increase in cell proliferation and enhanced TMEM16A-dependent secretion further enhanced the volume of M1 organoids (Fig. 4a, d). In contrast, organoid volume was significantly reduced in the presence of benzbromarone CaCCinhAO1, and Ani9, potent inhibitors of TMEM16A (Fig. 4d, Fig. S1). Moreover, both inhibitors blocked proliferation of M1 cells as measured in proliferation assays (Fig. 4e). M1 cells were grown as 2D cultures on permeable supports. Knockdown of PKD1 or PKD2 caused enhanced Cl^−^ secretion when stimulated by the Ca^2+^-dependent purinergic agonist ATP (Fig. 5a, c). Also, cAMP-dependent transport activated by IBMX and forskolin (IF) was augmented with knockdown of PKD1 or PKD2 (Fig. 5b, d). The data suggest that both Ca^2+^-activated TMEM16A and cAMP-dependent CFTR Cl^−^ channels contribute to renal cyst development. Co-staining of TMEM16A (green) and calreticulin (red) indicate upregulation of TMEM16A in plasma membrane and cytosol with knockdown of PKD1 or PKD2, while colocalization of TMEM16A with calreticulin was not observed (Fig. 5e, f).

### Disturbed Ca^2+^ signaling in PKD relies on TMEM16A

**Fig. 6:**
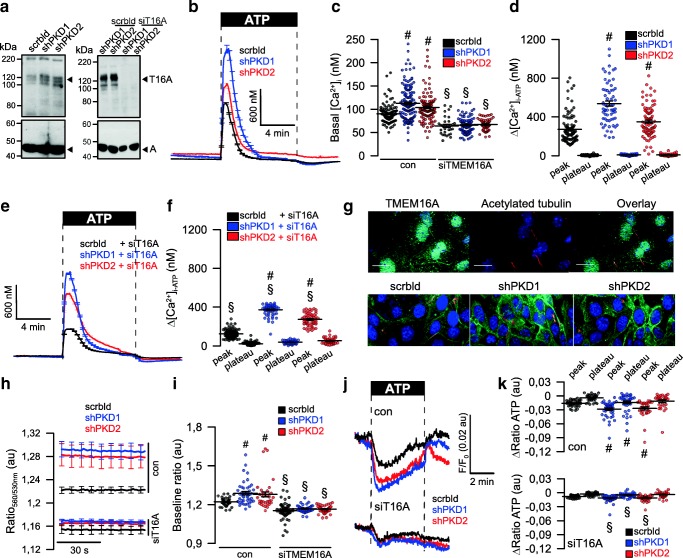
Upregulation of TMEM16A is essential for enhanced Ca^**2**+^ signaling upon knockdown of PKD1 and *PKD2*. **a** Left: Western blot indicating upregulation of TMEM16A by shRNA-knockdown of PKD1 or PKD2. Blots were performed *n* = 3 times. Protein loading was not normalized among the lanes. Densiometric analysis indicates an upregulation of TMEM16A by 1.6- (shPKD1) and 1.8 (shPKD)-fold. Right: siRNA-knockdown of TMEM16A upregulated by shRNA-knockdown of PKD1 or PKD2. Blots were performed in replicates. siRNA-knockdown of TMEM16A protein was 49 ± 3.8% (*n* = 5; western blotting) and 91 ± 8.5% (*n* = 3, real-time PCR). **b**–**d** Original recordings and summaries of basal Ca^**2**+^ and ATP (100 μM)-induced Ca^**2**+^ increase (Fura2) in control cells (scrbld), and cells with a knockdown of PKD1 or PKD2, respectively. **e**, **f** Original recordings and summaries of ATP-induced Ca^**2**+^ increase in cells lacking expression of TMEM16A (siT16A). **g** Expression of TMEM16A in M1 control cells (scrbld) and cells lacking expression of PKD1 or PKD2. **h**–**j** Original recordings and summaries of the effect of ATP on ER Ca^**2**+^ levels in control cells and cells lacking expression of PKD1 or PKD2. **k** Attenuated ATP-induced Ca^**2**+^ release after knockdown of TMEM16A. Bars = 20 μm. Mean ± SEM (number of monolayers measured). ^#^Significant difference when compared scrbld (*p* < 0.05; ANOVA). ^§^Significant difference when compared to control (*p* < 0.05; ANOVA)

Abrogated Ca^2+^ signaling in ADPKD has been intensely examined, but controversial results have been reported [6]. We reported a role of TMEM16A in Ca^2+^ signaling, i.e., enhanced agonist-induced Ca^2+^-store release by TMEM16A [14]. Here, we examined the role of TMEM16A for ER Ca^2+^-store release through IP_3_R and ryanodine receptors (RyR) upon knockdown of PKD1 and PKD2. shRNA-knockdown of PKD1 or PKD2 upregulated expression of TMEM16A (Fig. **6**a). Densiometric analysis indicates an upregulation of TMEM16A by 1.6 (shPKD1)- and 1.8 (shPKD)-fold. Knockdown of PKD1 or PKD2 enhanced basal [Ca^2+^]_i_ and augmented ATP-induced store release (Fig. **6**b–d). The enhanced Ca^2+^ signals observed in the absence of PKD1 or PKD2 required the presence of TMEM16A, as both basal Ca^2+^ levels and ATP-induced store release were strongly attenuated by siRNA-knockdown of TMEM16A, which was 49 ± 3.8% (*n* = 5) (Fig. 6a, c–f). Successful knockdown of TMEM16A was further validated by real-time RT-PCR and was 91 ± 8.5% (*n* = 3). Expression of TMEM16A is found in primary cilium and plasma membrane of polarized grown renal epithelial cells (Fig. **6**g, upper panel). Similar to M1-organoids, also M1-monolayers increase expression of TMEM16A upon knockout of PKD1 or PKD2 (Fig. **6**g, lower panel). Using the ER Ca^2+^ sensor ER-LAR-GECO1, we found higher basal ER Ca^2+^ levels and enhanced ATP-induced Ca^2+^ release in cells lacking expression of PKD1 or PKD2 (Fig. 6h–j). In contrast, knockdown of TMEM16A (Fig. **6**a) strongly reduced store filling and ATP-induced Ca^2+^-release (Fig. **6**k).

### Upregulated TMEM16A causes enhanced ER store release and store refill in ADPKD

**Fig. 7:**
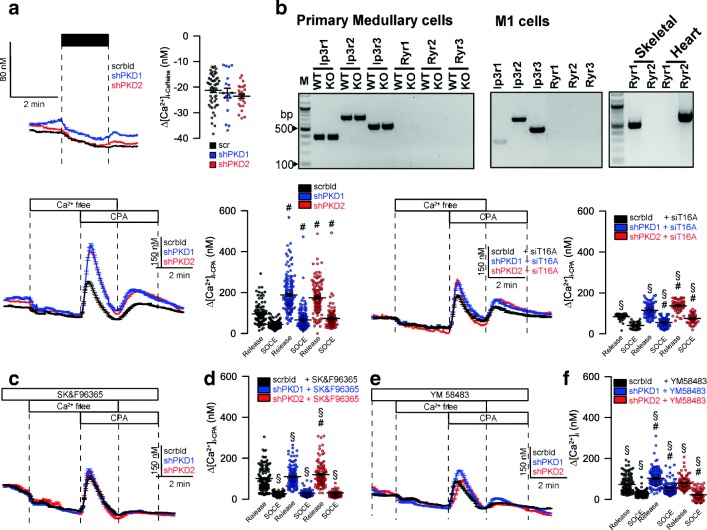
TMEM16A is essential for enhanced Ca^**2**+^ store release by knockdown of PKD1 and PKD2. **a**, **b** Lack of effects of caffeine on intracellular Ca^**2**+^ and lack of expression of RyR1–3 in mouse primary renal medullary and M1 collecting duct cells. **c**, **d** CPA (10 μM) induced store release in the presence or absence PKD1/PKD2. **e**, **f** CPA-induced store release was strongly attenuated by siRNA-knockdown of TMEM16A. **g**–**j** Original recordings and summaries of CPA-induced Ca^**2**+^ store release and SOCE in the presence of SK&F96365 and YM58483 (both 5 μM). Mean ± SEM (number of monolayers measured). ^#^Significant difference when compared scrbld (*p* < 0.05; ANOVA). ^§^Significant difference when compared to absence of siT16A or SK&F96365/YM58483, respectively (*p* < 0.05; ANOVA)

**Fig. 8:**
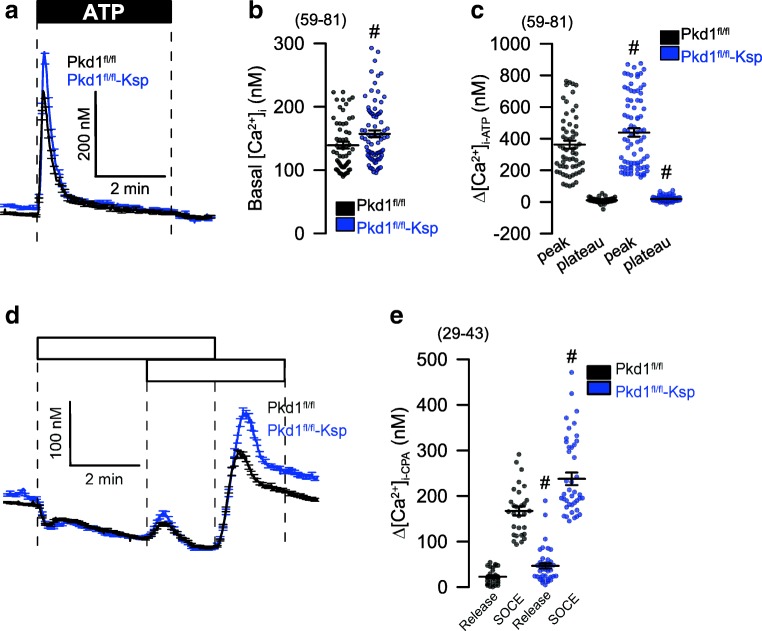
Enhanced Ca^**2**+^ signaling in primary renal tubular epithelial cells from PKD−/− mice. **a**–**c** Original recordings and summaries of basal Ca^**2**+^ and ATP (100 μM) induced Ca^**2**+^ increase (Fura2) in primary renal epithelial cells isolated from wild type mice (PKD^fl/fl^) and from mice with a renal tubular knockout of PKD1 (PKD^fl/fl^-KspCre). **d**, **e** Original recordings and summaries of Ca^**2**+^ increase induced by inhibition of SERCA with CPA (10 μM). Store release and store-operated Ca^**2**+^ influx (SOCE) was augmented in PKD^−/−^ cells. Mean ± SEM (number of cells measured). ^#^Significant difference when compared to wt (*p* < 0.05; ANOVA)

Ryanodine receptors were claimed to have a role in flow-induced Ca^2+^ increase in mouse kidney [3]. However, the activator of RyR, caffeine, did not increase intracellular Ca^2+^. Moreover, we did not detect expression of RyR1-3 in primary tubular epithelial cells from wild-type or PKD1−/− knockout animals, or M1 collecting duct cells (Fig. 7a, b). In contrast, signals for RyR1–3 were clearly present in skeletal muscle, heart muscle, and brain, respectively (not shown). Lack of PKD1 or PKD2 increased store emptying induced by inhibition of SERCA with cyclopiazonic acid (CPA). Moreover, store-operated Ca^2+^ entry (SOCE) was also enhanced by knockdown of PKD1/PKD2 (Fig. 7c, d). Enhanced store release and enhanced SOCE was strongly reduced in the absence of TMEM16A (Fig. 7e, f). Moreover, the inhibitor of transient receptor potential (TRP) channels SK&F96365 and the ORAI inhibitor YM58483 inhibited enhanced Ca^2+^ entry in PKD1/PKD2 knockout cells and abolished enhanced CPA-induced store release (Fig. 7g–j). Finally, to confirm the present results in M1 cells, we compared primary renal tubular epithelial cells from PKD^+/+^ and PKD1^−/−^ mice. We found enhanced ATP-induced Ca^2+^ store release, with a consecutive enhancement of store-operated Ca^2+^ influx (SOCE) in cells from PKD−/− animals, almost identical to the results obtained in M1 cells (Fig. 8). Taken together, the present data demonstrate augmented Ca^2+^ signals in the absence of either PKD1 or PKD2. These enhanced Ca^2+^ signals are caused by upregulation of TMEM16A Cl^−^ channels.

## Discussion

Aberrant intracellular Ca^2+^ signaling enhanced cell proliferation and fluid secretion are essential factors that drive growth of renal cysts [2]. Disturbed flow sensing and mechanical activation of Ca^2+^ influx into primary cilia were proposed as major mechanisms in ADPKD, a concept that has been questioned recently [17]. In the present paper, we found ATP-induced Ca^2+^ increase in both the primary cilium as well as in the cytosol near the plasma membrane of MDCK cells (Fig. 2). Although ciliary Ca^2+^ increase by ATP was larger, the responses in the cilium and the cytoplasm were similar. Both PKD1 and PKD2 are required for cellular trafficking and proper colocalization in the primary cilium of differentiated renal epithelial cells [3, 18]. Mutations or lack of expression of PKD2 may lead to compromised trafficking and accumulation of PKD1 in the ER, while defective or missing PKD1, loss of the primary cilium, or overexpression of PKD2 leads to enhanced density of PKD2 in the apical membrane and in the ER membrane [19]. Apart from localization within the primary cilium, both PKD1 and PKD2 have been detected at different subcellular locations (reviewed in [6]). In fact, most studies reported intracellular ER-localized PKD2. However, many studies have been performed in cultured cells under non-differentiated conditions. Moreover, it is notoriously difficult to obtain specific immunocytochemistry signals for the both proteins.

Inhibition of the IP_3_ receptors by PKD1, with attenuation of Ca^2+^ release from IP_3_-sensitive stores has been reported earlier [20]. Accordingly, receptor-mediated Ca^2+^ release will be enhanced with the loss of PKD1, as also observed in the present study. After proteolytic cleavage, a PKD1 fragment has been proposed to interact with the ER Ca^2+^-sensor STIM1 to inhibit store-operated calcium entry [21]. Lack of PKD1 therefore is likely to augment store-operated calcium entry, which is also shown here (Fig. 7). Enhanced Ca^2+^ entry was blocked by the inhibitor of receptor-mediated Ca^2+^ entry SK&F96365, and by the inhibitor of store-operated Orai1 Ca^2+^ influx channels, YM58483 (Fig. 7). Enhanced (and mislocalized) expression of PKD2 in the ER in the absence of PKD1 is likely to operate as a Ca^2+^-activated ER Ca^2+^ leakage channel, which will contribute to enhanced Ca^2+^ release from IP_3_-sensitive (IP_3_R) stores (Fig. 8). Notably, abnormal Ca^2+^ permeability of the ER membrane in ADPKD may account for both change in apoptotic activity and increased proliferation [16].

The present data are in line with the role of TMEM16A for intracellular Ca^2+^ signaling, which we reported earlier in cell models and transgenic animals [14]. TMEM16A channels enhance ER-Ca^2+^ store release by sequestering the ER and IP3 receptors to Ca^2+^ signaling compartments near the plasma membrane. However, in contrast to an earlier report [3], we were unable to detect any contribution of RyR channels in mouse primary renal epithelial cells or M1 cells (Fig. 7i, j).

The role of CFTR-dependent Cl^−^ secretion for growth of renal cysts has been implicated for long. We showed earlier that formation of cysts by principal-like MDCK cells is due to a synergism between cAMP and Ca^2+^-mediated fluid secretion [22]. The relevance of Ca^2+^-activated TMEM16A Cl^−^ channels became evident through our recent work [7, 23, 24]. Notably, STAT6-dependent transcription is upregulated in ADPKD [25]. Because expression of TMEM16A is also upregulated through activation of STAT6 (and STAT3), this could explain upregulation of TMEM16A in M1 cysts observed in the present study (Fig. 4a). Notably, TMEM16A supports proliferation, cell migration, and development of cancer by recruiting a number of intracellular signaling pathways [26]. Although the contribution of TMEM16A to ADPKD is not yet fully understood, the present data provide evidence for a pronounced impact on the disturbed intracellular Ca^2+^ signaling, caused by elimination of PKD1 or PKD2. As a number of potent inhibitors for TMEM16A are already available, TMEM16A may represent a novel drug target in the therapeutic regimen of polycystic kidney disease.

## Material and methods

*Cells*, *animals*, *virus production RT-PCR*, *complementary DNA* (*cDNA*): MDCK M2 and C7 cell lines were cultured in DMEM supplemented with 10% fetal bovine serum (FBS). M1 cells were cultured DMEM/F12 medium supplemented with 5% (v/v) fetal bovine serum (FBS), 1% insulin-transferrin-selenium 100x (ITS), and 1% l-glutamine 200 mM (all from Capricorn Scientific GmbH, Ebsdorfergrund, Germany) at 37 °C in a humidified incubator in 5% (v/v) CO_2_. M1 cells were transduced to downregulate Pkd1 and Pkd2. Mice with a floxed PKD1 allele were generously provided by Prof. Dr. Dorien J.M. Peters (Department of Human Genetics, Leiden University Medical Center, Leiden, The Netherlands) [27]. Experiments were approved by the local Ethics Committee of the Government of Unterfranken/Wuerzburg (AZ: 55.2–2532–2-328). Animals were euthanized between week 8 and 12, medullary tubular epithelial cells were isolated and were kept as primary culture. M1 cells were infected with lentiviral recombinant vectors containing the shRNAs of mouse Pkd1 (5´-GAATATCGGTGGGAGATAT) and Pkd2 (5´-GCATCTTGACCTACGGCATGA) with YFP_I152L_, as previously described [28, 29]. Stable transfected M1 cells were maintained in the presence of 5 μg/ml of Puromycin (Thermo Fisher Scientific, Darmstadt, Germany).

For semi-quantitative RT-PCR total RNA from M1 cells, MDCK cells and murine kidney were isolated using NucleoSpin RNA II columns (Macherey-Nagel, Düren, Germany). Total RNA (1 μg / 50 μl reaction) was reverse-transcribed using random primer (Promega, Mannheim, Germany) and M-MLV Reverse Transcriptase RNase H Minus (Promega, Mannheim, Germany). Each RT-PCR reaction contained sense (0.5 μM) and antisense primer (0.5 μM) (Table 1), 0.5 μl cDNA, and GoTaq polymerase (Promega, Mannheim, Germany). After 2 min at 95 °C, cDNA was amplified (30 cycles) for 30 s at 95 °C, 30 s at 57 °C, and 1 min at 72 °C. Real-time PCR of cDNA samples was performed in a LightCycler 480 device (Roche, Basel, Switzerland) using specific, intron-spanning primers (Table 2) and a SYBR® Green mastermix (Takyon, Eurogentec, Belgium). Target gene expression levels were quantified relative to beta-actin expression under consideration of PCR efficiencies calculated on the basis of standard dilution curves. The specificity of PCR amplifications was verified by agarose electrophoresis and melting curve analysis. PCR products were visualized by loading on peqGREEN (Peqlab; Düsseldorf, Germany) containing agarose gels and analyzed using ImageJ.

**Table 1:** RT-PCR primer (mouse)

Tmem16a (mouse)	Forward: 5′-GTGACAAGACCTGCAGCTAC	406 bp
	Reverse: 5′-GCTGCAGCTGTGGAGATTC	
Tmem16a (dog)	Forward: 5′-CTATAAGCTCCAGTCCCTAC	513 bp
	Reverse: 5′-CGACCCCGTGAATTTTAGTG	
Tmem16f (mouse)	Forward: 5′-CATACGAATCTAACCTTATCTGC	520 bp
	Reverse: 5′-CATTCTCTGTACAGGAGGTAAC	
Cftr (mouse)	Forward: 5′-GAATCCCCAGCTTATCCACG	544 bp
	Reverse: 5′-CTTCACCATCATCTTCCCTAG	
αEnac (Scnn1a, mouse)	Forward: 5′-CCTTGACCTAGACCTTGACG	409 bp
	Reverse: 5′-CGAATTGAGGTTGATGTTGAG	
βEnac (Scnn1b, mouse)	Forward: 5′-CAATAACACCAACACCCACG	588 bp
	Reverse: 5′-GAGAAGATGTTGGTGGCCTG	
γEnac (Scnn1g, mouse)	Forward: 5′-GCACCGACCATTAAGGACC	464 bp
	Reverse: 5′-GCCTTTCCCTTCTCGTTCTC	
Nkcc1(Slc12a2, mouse)	Forward: 5′-GCGAGAAGGTGCACAATAC	747 bp
	Reverse: 5′-CTGTACGGCTCGATCATGTC	
Pkd1 (mouse)	Forward: 5′-GTGGAAAGCAGGTCGGAAG	236 bp
	Reverse: 5′-TCGTCTCGTTCAGCACCAG	
Pkd2 (mouse)	Forward: 5′-GTGGATGTACACAAGTGAGAAGGAGC	454 bp
	Reverse: 5′-CACGACAATCACAACATCCAGACA	
Ptch1 (mouse)	Forward: 5′-GTCTTGGGGGTTCTCAATGGACTGG	590 bp
	Reverse: 5′-ATGGCGGTGGACGTTGGGTTCC	
Ptch2 (mouse)	Forward: 5′-GTGTGATCCTCACCCCGCTTGACTG	487 bp
	Reverse: 5′-CGCTCCAGCCGATGTCATGTGTC	
Gapdh (dog, mouse)	Forward: 5′-GTATTGGGCGCCTGGTCAC	200 bp
	Reverse: 5′-CTCCTGGAAGATGGTGATGG

**Table 2:** Primers for real-time PCR

Mouse Tmem16a	Forward: 5′-AGGAATATGAGGGCAACCTG	75
	Reverse: 5′-CGACACCATGGATTTTGGTA	
Mouse Pkd1	Forward: 5′-CATAGTGTGGAAAGCAGGTC	159
	Reverse: 5′-CAGTGACCCTCCAAGTACAC	
Mouse Pkd2	Forward: 5′-CTCAGGAGGAACTTCTGG	148
	Reverse: 5′-GAAACTGCCAAGAGGGTAC	
Beta-actin	Forward: 5′-CAACGGCTCCGGCATGTG	151
	Reverse: 5′-CTTGCTCTGGGCCTCGTC	

*Western blotting:* Protein was isolated from cells using a sample buffer containing 25 mM Tris–HCl, 150 mM NaCl, 100 mM dithiothreitol, 5.5% Nonidet P-40, 5% glycerol, 1 mM EDTA, and 1% protease inhibitor mixture (Roche, Mannheim, Germany). Proteins were separated by 7% sodium dodecyl sulfate (SDS) polyacrylamide gel and transferred to a polyvinylidene difluoride membrane (GE Healthcare Europe GmbH, Munich, Germany) or 4–20% Mini-PROTEAN TGX Stain-Free (Bio-Rad) using a semi-dry transfer unit (Bio-Rad). Membranes were incubated with primary anti-Tmem16a rabbit polyclonal antibody (Davids Biotech, Regensburg, Germany; 1:1000), anti-PKD1 (Polycystin-1 (7E12), Santa Cruz; 1:500) mouse antibody or anti-PKD2 (Polycystin-2 (D-3), Santa Cruz; 1:500) mouse antibody, overnight at 4 °C. Proteins were visualized using horseradish peroxidase-conjugated secondary antibody and ECL detection. Actin was used as a loading control. Equal amounts were loaded on each blot! Total protein on the blots was exactly the same under all conditions. Because the β-actin bands always show similar bands under all conditions and independent of the treatment with shPKD1, shPKD2, or siT16A (Figs. 4b and 6a), treatments do not change the expression of β-actin and this β-actin can be used as a reference point.

*M1 organoid model*: M1 cells were resuspended as a single-cell suspension in 50/50% Matrigel/type I collagen and transferred into 24-well plates (30 × 10^3^ cells/well, four wells per condition) for 9 days. Medium was changed every 3 days. Every 3 days, 30 random visual fields per well were photographed with an Axiovert 200 microscope (Zeiss, Germany). Cyst area of the lumina (~ 30–150 cysts per condition and single experimental procedure) was measured with AxioVision (Zeiss, Germany). Cyst volume was then estimated using the formula for the volume of a sphere, 4/3πr^3^.

*Immunocytochemistry*: M1 cells grown under confluent conditions for 4 days on glass coverslips and M1 organoids grown for 6 days were fixed for 10 min with methanol at − 20 °C. Organoids were isolated with ice-cold 5 mM EDTA in PBS and seeded in poly-l-lysine-coated coverslips. After seeding cells were fixed for 10 min with methanol at − 20 °C. After washing, cells were permeabilized with 0.5% (v/v, PBS) Triton X-100 for 10 min and blocked with 1% (w/v, PBS) bovine serum albumin for 1 h at room temperature. The cells were incubated overnight with primary antibodies (1:100) against rabbit anti-TMEM16A [30] (Davids Biotechnologie, Regensburg, Germany), rat anti-Ki-67 (DAKO, M7249, Germany), anti-mouse CFTR antibody ACL-006 (Alomone labs, Jerusalem, Israel), or mouse anti-acetylated tubulin (T7451, Sigma-Aldrich, Germany). Binding of the primary antibody was visualized by incubation with appropriate secondary antibodies conjugated with Alexa Fluor 488 or Alexa Fluor 546 (1:300, Molecular Probes, Invitrogen). Nuclei were stained with Hoe33342 (0.1 g/ml PBS, AppliChem, Darmstadt, Germany). Glass coverslips were mounted on glass slides with fluorescent mounting medium (DakoCytomation, Hamburg, Germany) and examined with an ApoTome Axiovert 200 M fluorescence microscope (Zeiss, Germany).

*Cell proliferation assay*: M1 cells were plated in 96-well plates at a density of 2 × 10^3^ cells per well for the time duration as indicated (0, 3, 6, and 9 days). Medium was changed every 3 days. Cells were incubated for 2 h in 100 μl of fresh media containing 0.5 mg/ml of the tetrazolium salt MTT. The dark blue formazan product was dissolved with DMSO and measured the absorbance at 595 nm.

*Ussing chamber*: MDCK or M1 cells were grown as polarized monolayers on permeable supports (Millipore MA, Germany) for 8 days. Cells were mounted into a perfused micro-Ussing chamber, and the luminal and basolateral surfaces of the epithelium were perfused continuously with Ringer’s solution (mmol/l: NaCl 145; KH_2_PO_4_ 0.4; K_2_HPO_4_ 1.6; glucose 5; MgCl_2_ 1; Ca^2+^ gluconate 1.3) at a rate of 5 ml/min (chamber volume 2 ml). Bath solutions were heated to 37 °C, using a water jacket. Experiments were carried out under open-circuit conditions. In addition, 100 μM ATP/UTP was added on the apical or basolateral side, or 100 μM 3-isobutyl-1-methylxanthine and 2 μM Forskolin (I/F) were added on the basolateral side, or 2 μM ionomycin was added on the apical side, as indicated in the figure. Data were collected continuously using PowerLab (AD Instruments, Australia). Values for transepithelial voltages (*V*_*te*_) were referred to the basolateral side of the epithelium. Transepithelial resistance (*R*_*te*_) was determined by applying short (1 s) current pulses (Δ*I* = 0.5 μA). *R*_*te*_ and equivalent short-circuit currents (*I*_*′SC*_) were calculated according to Ohm’s law (*R*_*te*_ *=* Δ*V*_*te*_*/*Δ*I*, *I*_*′SC*_ *= V*_*te*_*/R*_*te*_).

*Measurement of* [*Ca*^*2+*^]*i*: Primary cilium and membrane Ca^2+^ signals were detected after MDCK M2 and C7 cell were transfected with 5HT6-mCherry-GECO1.0 (5HT6-GECO, Addgene, Cambridge, MA, USA [31]). Cells were grown to confluence in glass coverslips and serum starved for 4–6 days to induce cilium formation. Afterwards, the cells were mounted and perfused in Ringer’s solution. The mCherry fluorescence of the indicator was used to localize the Ca^2+^ sensor. Therefore, before each experiment, a photo was taken exciting the 5HT6-GECO at 560 nm, and the emission was recorded between 620 ± 30 nm using a CCD-camera (CoolSnap HQ, Visitron Systems, Germany). To measure the ciliary Ca^2+^ changes, 5HT6-GECO was excited at 485/405 nm, and the emission was recorded between 535 ± 12.5 nm. The results for [Ca^2+^]cilium and [Ca^2+^]cyt were obtained at 485/405 nm changes and given in ratio. Measurement of the global cytosolic Ca^2+^ changes was performed as described recently [32]. In brief, cells were loaded with 5 μM Fura-2, AM (Molecular Probes) in OptiMEM (Invitrogen) with 0.02% pluronic (Molecular Probes) for 1 h at RT and 30 min at 37 °C. Fura-2 was excited at 340/380 nm, and the emission was recorded between 470 and 550 nm using a CCD-camera (CoolSnap HQ, Visitron Systems, Germany). Control of experiment, imaging acquisition, and data analysis were done with the software package Meta-Fluor (Universal imaging, USA). [Ca^2+^]_*i*_ was calculated from the 340/380 nm fluorescence ratio after background subtraction. The formula used to calculate [Ca^2+^]_*i*_ was [Ca^2+^]_*i*_ *= Kd* × (*R*-*R*_min_)/(*R*_max_-*R*) × (S_f2_/S_b2_), where *R* is the observed fluorescence ratio. The values *R*_max_ and *R*_min_ (maximum and minimum ratios) and the constant S_f2_/S_b2_ (fluorescence of free and Ca^2+^-bound Fura-2 at 380 nm) were calculated using 1 μmol/l ionomycin (Calbiochem), 5 μmol/l nigericin, 10 μmol/l monensin (Sigma), and 5 mmol/l EGTA to equilibrate intracellular and extracellular Ca^2+^ in intact Fura-2-loaded cells. The dissociation constant for the Fura-2·Ca^2+^ complex was taken as 224 nmol/l. ER Ca^2+^ signals were detected in Ca^2+^ sensor ER-LAR-GECO1 (Addgene, Cambridge, MA, USA [33],) expressing M1 cells. Cells were excited at 560 nm and emission was recorded between 620 ± 30 nm.

*Materials and statistical analysis*: All compounds used were of highest available grade of purity. Data are reported as mean ± SEM. Student’s *t* test for unpaired samples and ANOVA were used for statistical analysis. *p* < 0.05 was accepted as significant difference. We are grateful to Prof. Dr. Dorien J.M. Peters (Department of Human Genetics, Leiden University Medical Center, Leiden, The Netherlands) for providing us with animals with a floxed PKD1 allele. We acknowledge the support by Ms. Ana Fonseca.

## Electronic supplementary material

ESM 1 (PDF 80 kb).

ESM 2 (PDF 3998 kb).
