# Supplementary material for: TMEM16A drives renal cyst growth by augmenting Ca2+ signaling in M1 cells
Source: J Mol Med (Berl). 2020 Mar 18;98(5):659–71. doi: 10.1007/s00109-020-01894-y (PMC7220898; doi:10.1007/s00109-020-01894-y)
Supplement: Supplementary file 1 — (PDF 80 kb). [file 109_2020_1894_MOESM1_ESM.pdf]

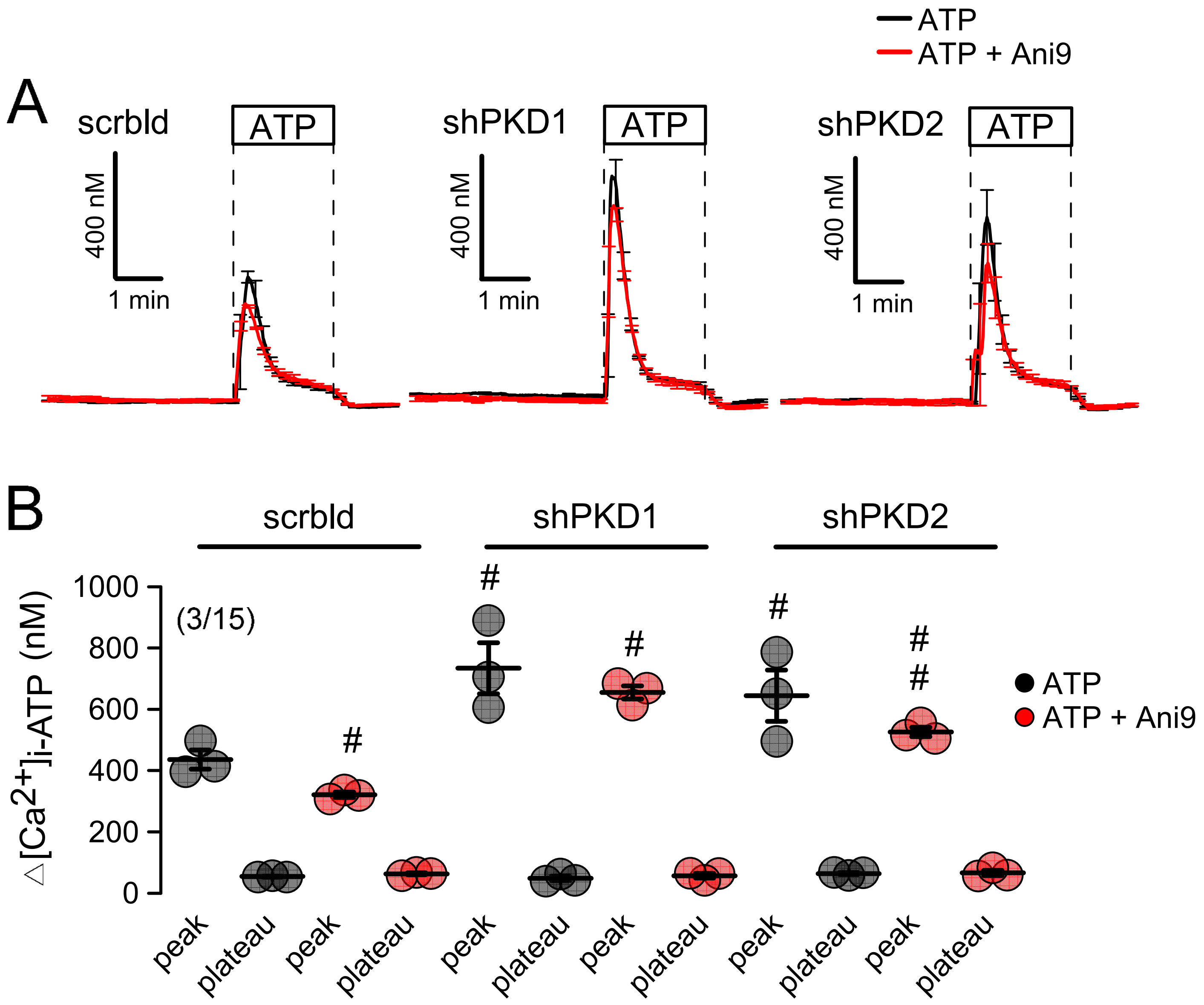

Fig. S1 *Inhibition of ATP-induced Ca^{2+} store release by Ani9***.** A) Original recordings of intracellular Ca^{2+} as measured by Fura2. ATP (100 µM) induced Ca^{2+} store release in M1 cells treated with shRNA for PKD1, shRNA for PKD2 or treated with scrambled RNA. Inhibition of store release by the TMEM16A inhibitor Ani9. B) Summary of store release and store operated Ca^{2+} influx. Mean ± SEM (number of cover slips/cells measured per cover slip). #significant difference compared to scrambled or absence of Ani9, respectively (p<0.5; ANOVA).
