# Supplementary figures and images for: TMEM16A drives renal cyst growth by augmenting Ca2+ signaling in M1 cells
Source: J Mol Med (Berl). 2020 Mar 18;98(5):659–71. doi: 10.1007/s00109-020-01894-y (PMC7220898; doi:10.1007/s00109-020-01894-y)

Fig. 1

A

M

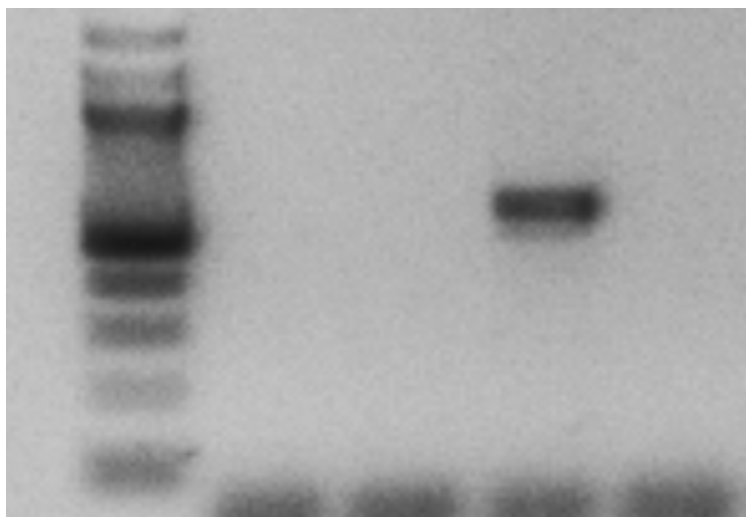

Fig. 3

A M

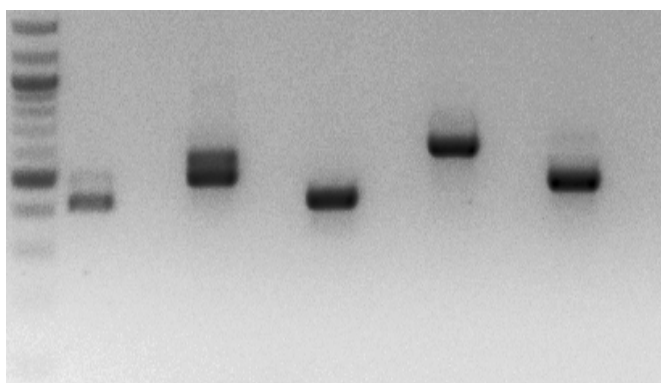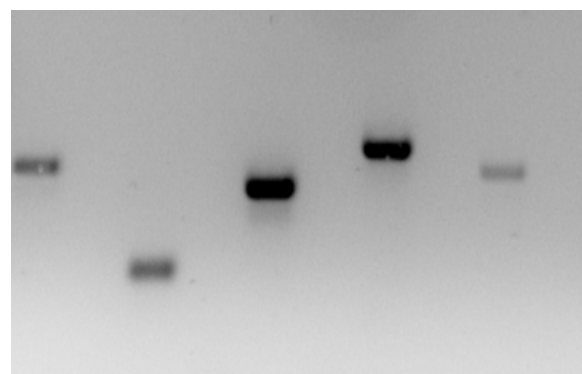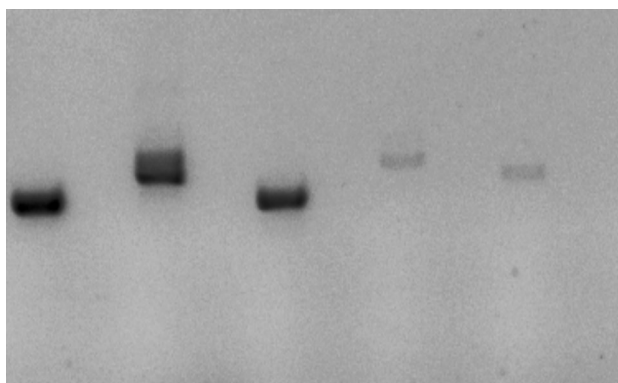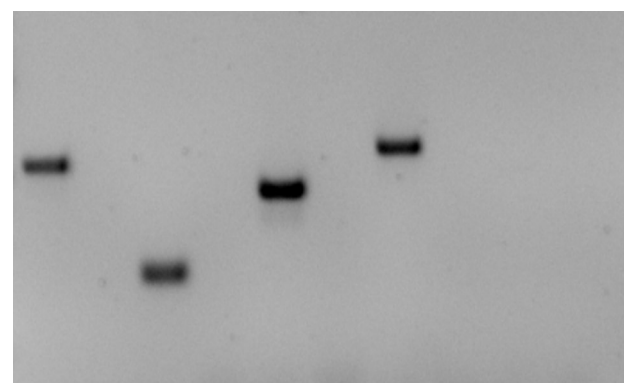

Fig. 4

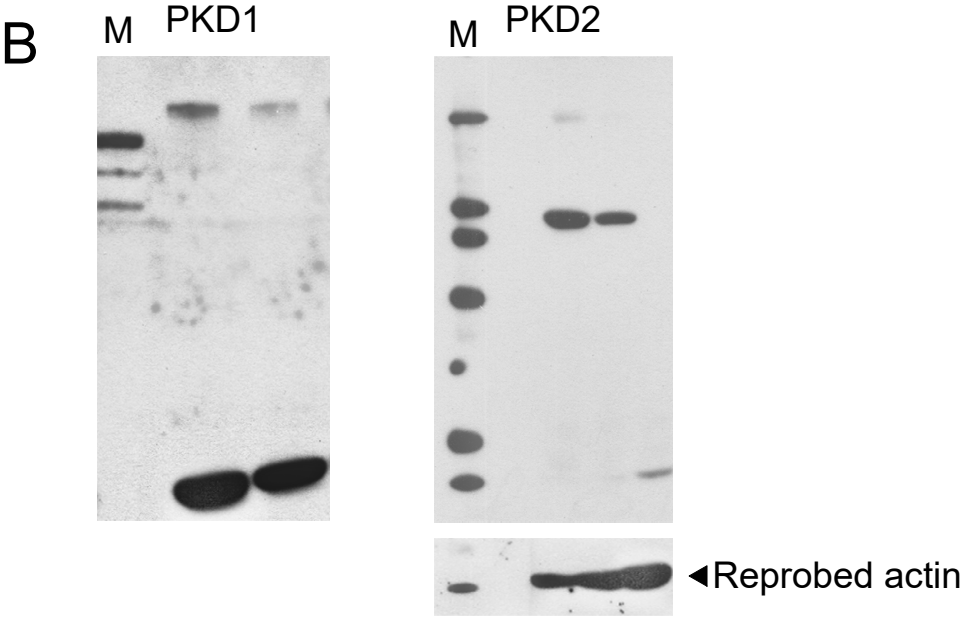

Fig. 6

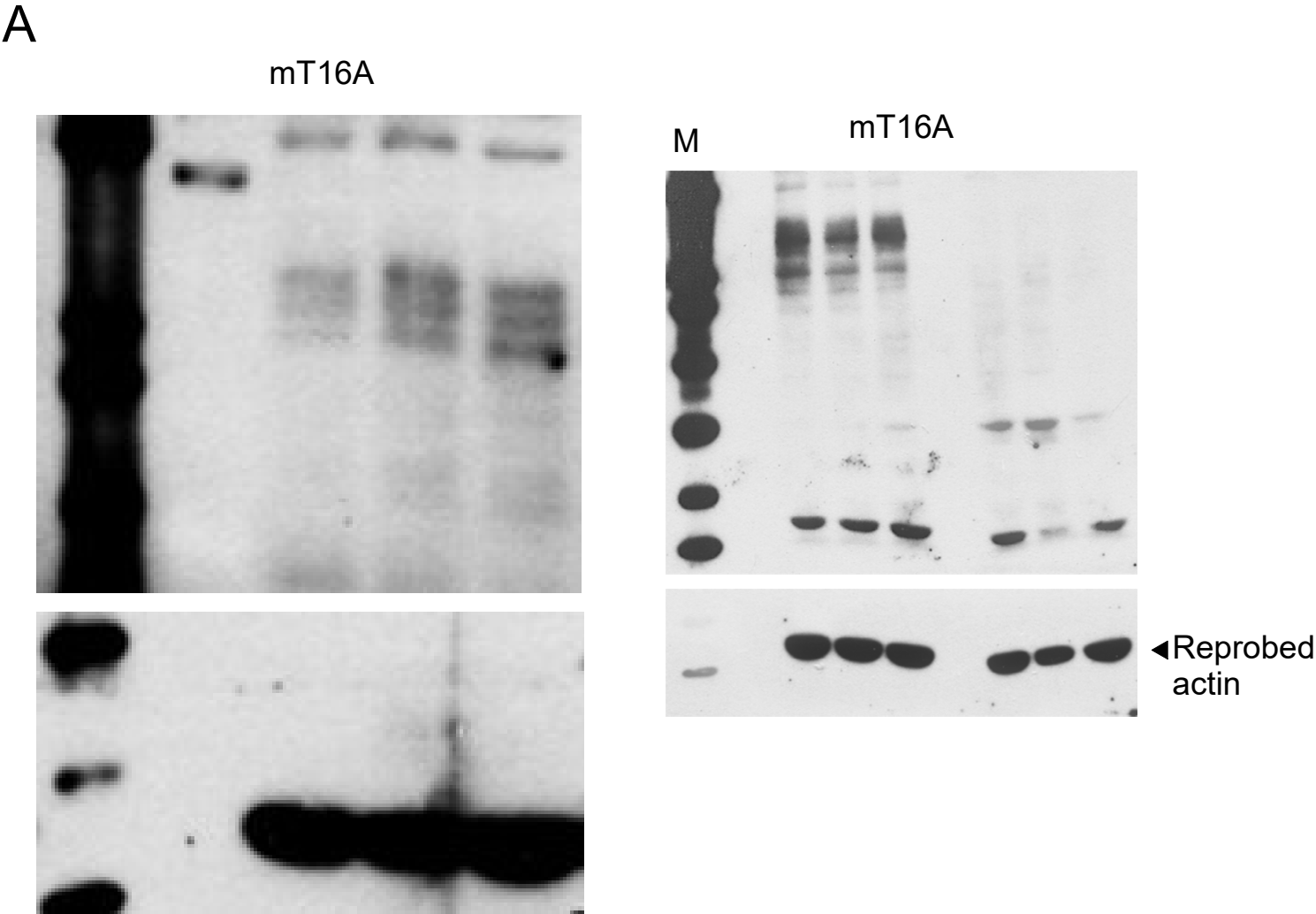

Fig. 7  
B

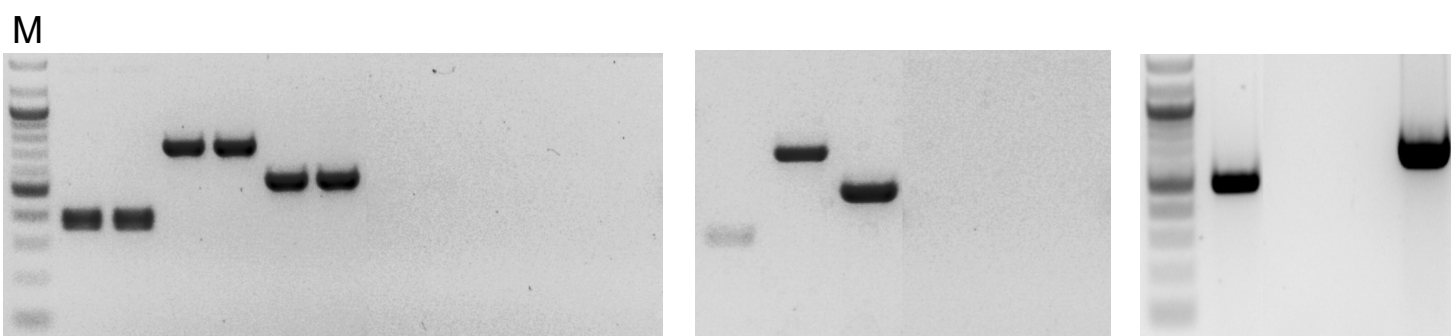

Supplement: Supplementary file 2 — (PDF 3998 kb). [file 109_2020_1894_MOESM2_ESM.pdf]
